# The Limits and Influences of a Nurse's Vocation

**Published:** 1888-01-14

**Authors:** R. Brudenell Carter

**Affiliations:** Member of the General Medical Council, Ophthalmic Surgeon to St. George's Hospital, and to the National Hospital for the Paralysed and Epileptic


### THE LIMITS AND INFLUENCES OF A NURSE'S VOCATION.

By R. BRUDENELL CARTER, F.R.C.S.,

Member of the General Medical Council, Ophthalmic Surgeon to St. George's Hospital, and to the National Hospital for the Paralysed and Epileptic. A Lecture introductory to a Course on Nursing, delivered to the Nurses of the Chelsea Infirmary.—(*Continued from p.* 246.)

WHEN I speak of a portion of decaying animal matter, you must not think that I mean a large portion. There was once a witness in a court of law who was asked a question about the size of something, and who said, after a great deal of deliberation, that it was about as large as a piece of chalk. I suppose we may venture to guess that he meant a piece such as it would be convenient to hold in the hand for making marks with; and our decaying matter need not be as large as a piece of chalk. In the ward to which I have referred it could only have found access in the form of dust; and it would be far more dangerous in the form of dust than if introduced in larger pieces, because it would be more easily carried about in currents of air, and would more readily find its way wherever the air itself could penetrate. The noxious influence which it would exert is more a matter of quality than of quantity, and a very small quantity of harmful dust may be sufficient to bring the best hopes and best endeavours of doctor and nurse to disappointment. Think for a moment what the dust of a London street is made of. It is dried mud, and the mud is composed of the pieces worn away from the pavement, and from horse-shoes and the wheels of vehicles, by traffic, mixed with dead cats, fishes' heads and entrails, the droppings of animals, and every other conceivable abomination. Even here in this infirmary, in a street of comparatively small traffic, it will help you to realise the character of the dust if you smear a slip of glass with glycerine, and place it in an open window. The dust will stick to it, and I am sure that one of the doctors would, if you were to ask him, put it under a microscope, and let you see what the dust was composed of. Besides pieces of the various things I have mentioned, you would see scales of human skin, portions of hair, and many other kinds of animal and therefore noxious refuse. If this sort of stuff is, as we have seen, poisonous when it gains access to the surfaces of wounds, we cannot think that it is beneficial, or indeed otherwise than hurtful, when it is breathed or swallowed; although, in the latter cases, its effects may not be so soon or so plainly declared. Still, it is always an enemy, and one of the first principles of nursing is to strive against it. In order to strive effectually, it is necessary to remove from the sick-room everything by which dust is harboured, especially carpets and curtains, as far as this is possible. If you want to know how much dirt there is in a carpet, set two or three children to have a game of romps in a room with a carpeted floor; and, when they have finished, go into the room half an-hour afterwards, and look at the surfaces of the tables. As for window or bed curtains, when they have been hanging in London for a short time, you could take up the dirt in their folds in a teaspoon. Every time you walk over the carpet, and, in a still greater degree, every time the curtains are drawn or undrawn, a cloud of dust issues, and floats about in the air to settle somewhere at last. Besides carpets and curtains, all superfluous furniture should be removed, and especially all woollen or silken table-covers, to which dirt would stick. When all harbour is thus, as far as may be, got rid of, the necessary dusting of the articles which remain should be done with a damp cloth, to which dust will stick, and by which it will be carried away, and never with a dry one, which only sets the dust in movement. The floor and the walls should also be wiped with a damp cloth; and the chances of infection from external dirt will in this way be rendered as small as possible. Those of you who have the advantage of nursing in public institutions have all these precautions taken for you; and it is only when you come into private families that you will discover the necessity of insisting upon them or of carrying them out. And here I may remark that the cleaning of a room does not properly form part of a nurse's work, and ought to be done for her, under her direction; but, if you cannot get it done to your entire satisfaction, you should never hesitate for a moment to do it yourselves. Making a bed can hardly be called part of a doctor's work; but yet, in old times, when there were no trained nurses, and I found a patient on a badly-made and uncomfortable bed, I never hesitated to make the bed myself, and to have up the women of the house in order to teach them how to do it properly. The first object of both doctor and nurse is to obtain the recovery of the patient, and neither one nor the other can afford to throw away a chance. Both alike ought to be capable of doing, and ready to do, if necestary, everything which can be required in the sick-room, and should never be compelled to look helplessly around in order to find someone else to do it for them. If they are compelled so to look around, the patient will probably die while they are looking; or at least will be like that King of Spain who was badly burnt while the Lord Chamberlain was asking the first Lord in Waiting to request the second Lord in Waiting to desire the Gentleman Usher of the Black Rod to order the groom of the chambers to tell the page to direct two footmen to come and move his Majesty's chair a little farther away from the fire. In view of her responsible duties, a nurse should never overtax her strength in any way which can be avoided, and should avail herself of all the efficient help which she can obtain; but at the same time, she should know exactly what ought to be done in a sick-room, and should be prepared, if she cannot get it done by others, to do it herself rather than allow her patient to suffer.

I pass on from the cleanliness of the room to the cleanliness of the patient; and here what I have said about the poisonous character of dirt applies with double force. In the mere act of living, we use up certain portions of the fabric of the body, and the waste which is thus formed, and which in health is removed as fast as it is formed, either in the expired breath, by the kidneys, by the bowels, or by the skin, is fatally poisonous if retained. Go into a bed-room, especially an unventilated bed-room, in which a person with dirty skin has passed the night, and your nose will enable you to form some idea of the character of the material which it is the business of the skin to remove from the body. You will also, I think, be in no doubt as to the necessity of cleaning away this material from the surface on which it is left; and you will understand why perfect personal cleanliness, such as is obtained by frequent washing or sponging of the surface of the skin, and frequent renewal of bed linen and body linen, is so essential in many forms of illness. It will be a part of your training to learn how to accomplish the washing of the patient without doing harm; and, moreover, this question of personal cleanliness is one of those in which it will sometimes happen that the general principle has to be modified in exceptional cases. Especially in typhoid fever there are stages of the disease in which the doctor has sometimes to forbid the ordinary washing, because even the slight bodily movement required for it might prove fatal to the patient.

Proceeding next to the question of ventilation, let me remind you that, just as water washes away dirt from the surface of the body, so constantly renewed air washes away the dirt which is expelled from the lungs. The smell of the unventilated bed-room of the dirty person, to which I have already alluded, is partly due to what is thrown off by the skin, but partly, also, to what is thrown off by the lungs. If you neglect ventilation, you compel the patient to breathe over again, and to take once more into his system, that which he has just been at the greatest pains to remove. His state is something like that of a person who is trying to keep his hands clean, in the course of dirty work, by washing them over and over again in the same basin of water. Apart from this, it is to be borne in mind that air once breathed is spoiled for respiratory purposes; and that its renewal is absolutely necessary in order to enable it to fulfil its natural office of renovating the blood, so that this may carry the material called oxygen—which fresh air contains in a state of activity, and which is essential to the maintenance of life—to every part of the body. The patient who is condemned to breathe air which has been deprived of an important part of its oxygen, and which has become loaded with the products of previous respiration, is being subjected to a process of gradual suffocation, a process which would be highly injurious in health, and which is often fatal in disease. It will be an important part of your professional knowledge that you should be acquainted with the value and necessity of fresh air, and also with the best means of introducing it into the sick-room. Speaking generally, open windows are the only effectual channels, and the greatest care should be taken to exclude air which has come through stuffy passages and the like, or which has been exposed to any possibilities of domestic contamination on its way. Bath-rooms, closets, lavatories, and all places which contain waste pipes or sinks, are liable to admit foul air by the channels which are provided for the escape of dirty water; and it is necessary to take care, when rooms so fitted open into bed-rooms, that the door of communication should be kept closed, so that no air of questionable character may be admitted. At the same time, the opening of windows requires circumspection in cold weather, and should only be done when the patient is thoroughly protected against undue chilling of the surface of the body. With sufficient coverings, and, if necessary, with a fire, the window of a sick-room may always be open, to some extent, both by night and by day. The fire itself is an important adjunct to ventilation, since it affords a steady outlet through the chimney, and thus provides for the removal of the air which is vitiated, in order to make room for the entrance of that which is fresh.

*(To be continued.)*

